# Trajectories of Sleep Duration, Sleep Onset Timing, and Continuous Glucose Monitoring in Adults

**DOI:** 10.1001/jamanetworkopen.2025.0114

**Published:** 2025-03-05

**Authors:** Luqi Shen, Bang-yan Li, Wanglong Gou, Xinxiu Liang, Haili Zhong, Congmei Xiao, Ruiqi Shi, Zelei Miao, Yan Yan, Yuanqing Fu, Yu-ming Chen, Ju-Sheng Zheng

**Affiliations:** 1 Westlake Laboratory of Life Sciences and Biomedicine, Hangzhou, China; 2 Guangdong Provincial Key Laboratory of Food, Nutrition and Health, Department of Epidemiology, School of Public Health, Sun Yat-sen University, Guangzhou, China; 3 Affiliated Hangzhou First People’s Hospital, School of Medicine, Westlake University, Hangzhou, China; 4 Institute of Basic Medical Sciences, Westlake Institute for Advanced Study, Hangzhou, China; 5 Research Center for Industries of the Future, School of Life Sciences, Westlake University, Hangzhou, China

## Abstract

**Importance:**

Understanding the interplay between trajectories of sleep duration, sleep onset timing, and glycemic dynamics is crucial for improving preventive strategies against diabetes and related metabolic diseases.

**Objective:**

To examine the associations of sleep duration and onset timing trajectories with continuous glucose monitoring (CGM)–derived glycemic metrics in adults.

**Design, Setting, and Participants:**

This cohort study analyzed data collected from January 2014 to December 2023 in the Guangzhou Nutrition and Health Study, a prospective cohort in Guangdong province, China, among participants aged 46 to 83. Participants who had repeated sleep assessments at several study visits and were equipped with CGM devices at the last visit were included. Data analyses were conducted between January and June 2024.

**Exposures:**

The trajectories of sleep duration and onset timing were constructed using self-report sleep duration and sleep onset timing, recorded at multiple study visit points.

**Main Outcomes and Measures:**

Measurements of glycemic variability and glycemic control were collected using a masked CGM device worn by patients for 14 consecutive days. Huber robust regression models were used to assess the associations between sleep trajectories and CGM-derived metrics.

**Results:**

In this study of 1156 participants (mean [SD] age, 63.0 [5.1] years, 816 [70.6%] women), we identified 4 distinct sleep duration trajectory groups: severe inadequate, moderate inadequate, mild inadequate, and adequate. Severe sleep inadequacy was associated with an increment of glycemic variability indicators: 2.87% (95% CI, 1.23%-4.50%) for coefficient of variation and 0.06 (95% CI, 0.02-0.09) mmol/L for mean of daily differences. We found 2 trajectories of sleep onset timing: persistent early and persistent late groups. Late sleep onset was associated with larger coefficient of variation (β = 1.18%; 95% CI, 0.36%-2.01%) and mean of daily differences (β = 0.02 mmol/L; 95% CI, 0.01-0.04 mmol/L). Inappropriate sleep duration and timing trajectories in combination were associated with greater glycemic variability.

**Conclusions and Relevance:**

In this cohort study of middle-aged and older participants, persistent inadequate sleep duration and late sleep onset, whether alone or in combination, were associated with greater glycemic variability. These findings emphasize the importance of considering both sleep duration and timing for optimizing glycemic control in the general population.

## Key Points

**Question:**

Are distinct sleep status trajectories associated with differential glycemic dynamics in the general population?

**Findings:**

In this cohort study of 1156 participants aged 46 to 83, the trajectories of long-term insufficient sleep duration and persistent late sleep onset, whether alone or in combination, were associated with greater fluctuations in blood glucose levels as monitored by continuous glucose monitoring.

**Meaning:**

These findings provide important evidence supporting the health benefit of sufficient sleep duration and early sleep onset for optimizing glycemic control in adults.

## Teaser

This cohort study evaluates the associations of sleep duration and onset timing trajectories with continuous glucose monitoring–derived glycemic metrics in adults in China.

## Introduction

Sleep serves as a fundamental component of daily life, playing a pivotal role in maintaining human health.^1^ Suboptimal sleep status, such as inadequate duration, diminished quality, and late timing, are known to impair metabolic homeostasis, notably affecting glucose metabolism.^2^ While previous research has attempted to establish a connection between sleep and fasting blood glucose levels, there has been less focus on the understanding of glycemic dynamics and fluctuation in the general population. Given the critical role of glycemic variability in diabetes management and its significant implications for long-term health outcomes,^3,4,5,6,7,8,9^ deciphering the relationship between sleep and glycemic dynamics could inform better preventive strategies for diabetes and related metabolic diseases.

The advent of continuous glucose monitoring (CGM) devices has provided a good opportunity to gain a comprehensive view of blood glucose dynamics over extended periods.^10^ Several cross-sectional studies have demonstrated the associations between sleep measures and CGM-derived glucose profiles in a general population^11^ or in patients with type 2 diabetes (T2D).^12^ Nevertheless, the trajectories of long-term sleep patterns with subsequent dynamics of glycemic metrics as captured by CGM devices are still unclear.

Therefore, the present study aimed to investigate the associations of long-term sleep duration and onset timing trajectories with CGM-derived glycemic dynamics in 1156 middle-aged and elderly Chinese participants. In addition, we aimed to evaluate the joint effects of sleep duration and onset timing on glycemic control, with the hypothesis that the poorest glycemic control would be observed in individuals experiencing both short sleep duration and later sleep onset.

## Methods

### Study Design and Population

Our study adhered to the Strengthening the Reporting of Observational Studies in Epidemiology (STROBE) reporting guideline for cohort studies. Before to participation, written informed consent was obtained from all individuals. Ethical approval for the study protocol was granted by the ethics committees of the School of Public Health at Sun Yat-sen University and Westlake University. The present study was conducted based on the Guangzhou Nutrition and Health Study (GNHS), an ongoing community-based prospective cohort in Guangdong province, China.^13^ The GNHS included Chinese participants aged 40 to 75 years who had been residents of Guangzhou for at least 5 years at the inception of the study in 2008. Follow-up assessments were systematically conducted every 3 years.

The follow-up visit between 2014 and 2017 of the GNHS cohort served as the baseline for the current analyses, as sleep duration data collection commenced at this point. Among 2345 participants with sleep data at baseline (ie, during the follow-up between 2014 and 2017), 1300 were equipped with a CGM device after around 6 years of follow-up, all of whom had valid CGM data. We excluded 144 participants due to the lack of repeated sleep duration assessment or invalid sleep records (104 participants) or missing baseline characteristics (40 participants). After these exclusions, 1156 individuals were included in the sleep duration analyses (eFigure 1 in Supplement 1). A total of 1109 participants were included in our longitudinal analysis of sleep onset timing trajectories with CGM-derived metrics (eFigure 1 in Supplement 1).

### Extraction of CGM-Based Glycemic Metrics

Participants wore a masked CGM device (Freestyle Libre H, Abbott Diabetes Care Inc) for 14 consecutive days, capturing interstitial glucose every 15 minutes. To ensure data accuracy, CGM readings were validated by excluding recordings (1) from the first and last days of incomplete CGM connection; (2) spanning less than 72 hours; and (3) from days with extreme time below range (>99th percentile). The remaining recordings were considered valid and used for the following analyses.

We selected coefficient of variation (CV), mean amplitude of glycemic excursions (MAGE), mean of daily differences (MODD), and SD as representative measures of glycemic variability. CV was determined by dividing SD by mean glucose values to reflect relative variability.^14^ MAGE, the mean glucose amplitude exceeding 1 SD within a 24-hour period, quantified intraday variability.^15^ MODD, the mean of the absolute differences between glucose values at the same time on different days, capture interday variability.^16^

Time in range (TIR) and mean blood glucose (MBG) were used as clinically validated indices of current glycemic control. TIR quantified the percentage of time glucose levels remained within the target glucose range of 3.9 to 10 mmol/L over 24 hours.^17^ Daily MBG was computed by averaging 24-hour blood glucose level across the monitoring periods for each participant.^18^

### Assessment of Sleep Parameters

#### Sleep Duration

Sleep duration was assessed at 3 time points: baseline (2013-2017), first follow-up (2017-2021), and second follow-up (2021-2023). At each visit, participants reported their typical daily sleep duration over the past month using an open-ended questionnaire. This approach enabled the examination of sleep duration trajectories across the 3 time points.

#### Sleep Onset Timing

Sleep onset timing was assessed during 2 follow-up visits, first follow-up (2017-2021) and second follow-up (2021-2023), respectively. Participants were asked to report their typical daily bedtime and the duration it took them to fall asleep (sleep latency) over the past month. We evaluated the trajectories of sleep onset timing over 2 visits.

### Statistical Analyses

All analyses were performed with SAS version 9.4 (SAS Institute) or R version 4.1.2 (R Project for Statistical Computing). To identify trajectory patterns of sleep duration and sleep onset timing over the course of the study, we used group-based trajectory modeling.^19,20,21^ The optimal number of trajectories was determined using a combination of the Bayes Information Criterion, sample size considerations, scientific plausibility, and existing literature.^21^ We first considered 2, 3, 4, or 5 trajectories for each sleep feature (sleep duration and sleep onset timing) and finally discerned 4 distinct trajectory groups for sleep duration and 2 groups for sleep onset timing, incorporating participants with valid sleep data into these trajectories. We then calculated the posterior probability for each participant of being a member of each of the sleep feature trajectory groups and assigned participants to the trajectory group with the greatest posterior probability of membership. The mean posterior probability for all trajectory groups exceeded 0.70, and each trajectory group composed more than 5% of the participants (eTable 1 in Supplement 1), suggesting high internal reliability and sufficient discrimination among individuals with varying sleep feature patterns.

Considering the distribution of CGM-defined glycemic control metrics, Huber robust regression models^10,11^ were used to assess the associations between the identified sleep duration trajectory groups and CGM-derived mean-glucose level and glycemic variability. A parallel analysis was conducted for sleep onset timing trajectory groups. Participants were further classified into combined sleep duration and sleep onset timing groups, resulting in 6 distinct groups. The association between these combined groups and glycemic variability was then evaluated using Huber robust regression models. Covariates included in model 1 were age and sex. Model 2 considered the additional covariates of body mass index (BMI), baseline education level, household income, smoking status, alcohol consumption status, physical activity, total energy intake, and tea and coffee consumption. Model 3 included covariates in model 2, with the addition of hemoglobin A_1c_ concurrent with the first time of sleep data collection. Details of covariate measurements are available in the eMethods in Supplement 1. Huber robust regression models were used to assess the associations between the identified sleep onset timing trajectory groups and CGM-derived mean glucose level and glycemic variability. *P* < .05 was considered statistically significant. To account for multiple comparison, a Benjamini-Hochberg false discovery rate (FDR) of 5% was applied. FDR < 0.05 was considered statistically significant.

To ascertain the robustness of our findings, we performed several sensitivity analyses based on the previously described model 2. First, we incorporated snoring, daytime sleepiness, and sleep apnea–related disease conditions (hypertension, T2D, and dyslipidemia) as additional covariates to evaluate potential confounding effects. Second, an alternative referent, the mild inadequate group for sleep duration was employed to determine if the associations held when compared with a different reference group. Third, the analytical focus was shifted from sleep trajectory groups to the baseline assessment of sleep duration and sleep onset timing. Finally, we accounted for the mutual adjustment in sleep duration and sleep onset timing trajectories when examining the associations between sleep patterns and CGM-derived metrics.

## Results

### Characteristics of Study Participants

A total of 1156 participants (mean [SD] age of 63.0 [5.1] years at baseline; 816 women [70.6%]) were included in the analyses of our present study (Table 1). Participants reported a mean (SD) of 6.9 (1.2) sleeping hours per night at baseline, with 795 participants (71.7%) sleeping before midnight during the first follow-up. Sleep duration slightly decreased to 6.6 hours and 6.4 hours during the first and second follow-ups, while sleep onset timing remained similar between these 2 time points (Table 1). The mean (SD) values were 24.3% (5.7%) for CV, 1.5 (0.5) mmol/L for SD, and 3.6 (1.3) mmol/L for MAGE. The median (IQR) for MODD was 0.2 (0.19-0.35) mmol/L. Baseline characteristics of the study participants are presented in Table 1. There were no demographic differences between the participants included and those excluded in our study (eTable 2 in Supplement 1).

**Table 1.** Characteristics of Study Participants in Guangzhou Nutrition and Health Study

Characteristic	Participants, No (%)		
	Overall (N = 1156)	Women (n = 816)	Men (n = 340)
Age, mean (SD), y	63.0 (5.1)	62.3 (4.5)	64.6 (6.1)
Body mass index, mean (SD)^a^	23.4 (3.3)	23.1 (3.2)	24.0 (3.4)
Current smoker	63 (5.4)	1 (0.1)	62 (18.2)
Current alcohol drinker	97 (8.4)	35 (4.3)	62 (18.2)
Tea consumption	600 (51.9)	357 (43.8)	243 (71.5)
Education			
Middle school or lower	266 (23)	191 (23.4)	75 (22.1)
High school or professional college	541 (46.8)	420 (51.5)	121 (35.6)
University	349 (30.2)	205 (25.1)	144 (42.4)
Household income, Chinese ¥/mo/person^b^			
Extremely low (≤500)	21 (1.8)	13 (1.6)	8 (2.4)
Low (501-1500)	276 (23.9)	203 (24.9)	73 (21.5)
Middle (1501-3000)	539 (46.6)	400 (49.0)	139 (40.9)
High (>3000)	320 (27.7)	200 (24.5)	120 (35.3)
Physical activity, mean (SD), MET-h/d	42.1 (15.1)	42.5 (15.0)	41.1 (15.4)
Total energy intake, mean (SD), kcal/d	1800.8 (564.4)	1735.2 (532.9)	1958.4 (606.1)
Hypertension	397 (34.3)	247 (30.3)	150 (44.1)
Dyslipidemia	646 (55.9)	461 (56.5)	185 (54.4)
Type 2 diabetes	131 (11.4)	79 (9.8)	52 (15.4)
Sleep duration at baseline, mean (SD), h	6.9 (1.2)	6.8 (1.2)	7.1 (1.2)
Sleep duration at first follow-up, mean (SD), h	6.6 (1.3)	6.5 (1.3)	6.8 (1.2)
Sleep duration at second follow-up, hours, mean (SD)	6.4 (1.2)	6.3 (1.3)	6.6 (1.2)
Sleep onset timing before midnight during first follow-up	795 (71.7)	554 (71.4)	241 (72.4)
Sleep onset timing before midnight during second follow-up	802 (72.3)	550 (70.9)	252 (75.7)
Hemoglobin A_1c_, mean (SD), %	6.0 (0.8)	6.0 (0.7)	6.1 (0.8)
CGM-derived metrics			
Daily MBG, mean (SD), mg/dL	107.0 (24.9)	106.9 (23.2)	107.2 (28.5)
TIR, median (IQR), %	94.0 (86.2-97.3)	94.4 (87.1-97.4)	92.8 (83.6-97.1)
CV, mean (SD), %	24.3 (5.7)	23.9 (5.3)	25.2 (6.6)
SD, mean (SD), mg/dL	26.4 (9.8)	26.0 (9.3)	27.3 (10.7)
MAGE, mean (SD), mg/dL	64.5 (23.9)	63.6 (22.8)	66.5 (26.3)
MODD, median (IQR), mg/dL	4.4 (3.3-6.3)	4.3 (3.3-6.9)	4.7 (3.5-6.9)

Abbreviations: CGM, continuous glucose monitor; CV, coefficient of variation; MAGE, mean amplitude of glucose excursions; MBG, mean blood glucose; MET, metabolic equivalent of task; MODD, mean of daily differences; TIR, time in range.

SI conversion factors: To convert glucose to mmol/L, multiply by 0.0555; hemoglobin A_1c_ to proportion of total hemoglobin, multiply by 0.01.

^a^ Calculated as weight in kilograms divided by height in meters squared.

^b^ Conversion rate: 1 Chinese ¥ = $0.14 USD.

### Sleep Duration Trajectories and Their Associations With CGM-Derived Glycemic Control Metrics

**Figure 1.**
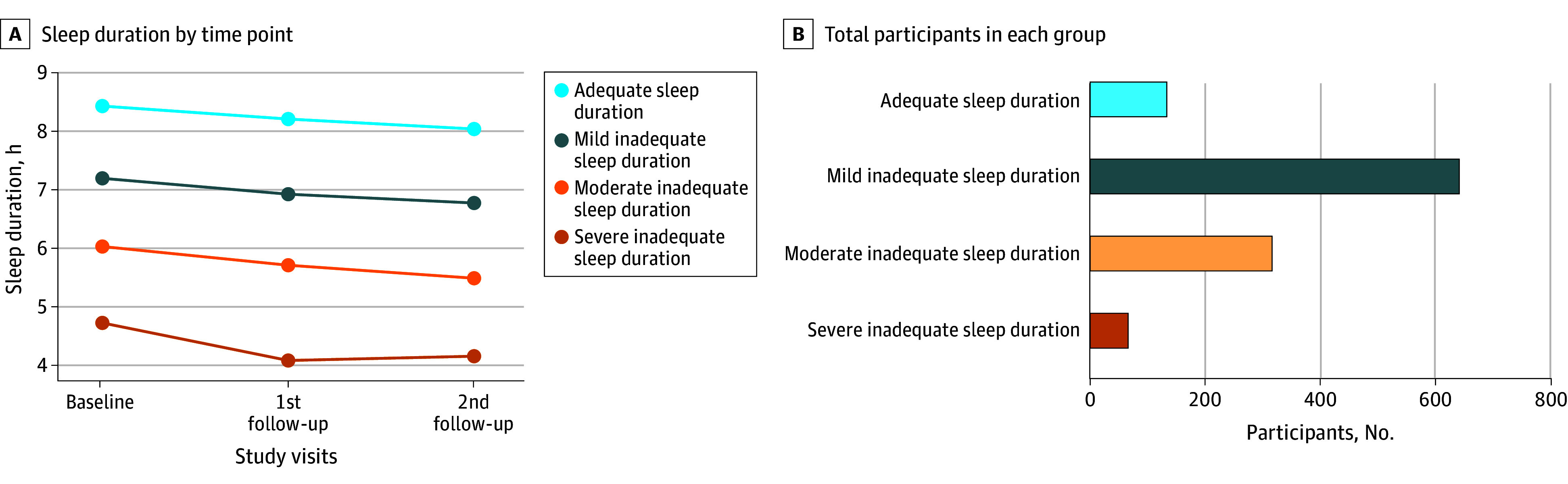
Description of Sleep Duration Trajectories at Baseline, First Follow-Up, and Second Follow-Up The latent class growth modeling, a group-based modeling approach (SAS Proc Traj), was used to identify subgroups that shared a similar sleep duration trajectory from baseline to follow-up visits. The model with 4 trajectories showed the best fit to the data, including a severe inadequate group, a moderate inadequate group, a mild inadequate group, and an adequate group.

We identified 4 distinct trajectories of sleep duration within the study population: severe inadequate (66 participants [5.7%] from 4.7 to 4.1 hours per night), moderate inadequate (316 participants [27.3%] from 6.0 to 5.5 hours per night), mild inadequate (641 participants [55.4%] from 7.2 to 6.8 hours per night), and adequate (133 participants [11.5%] from 8.4 to 8.0 hours per night) (Figure 1; eFigure 2 in Supplement 1).

**Figure 2.**
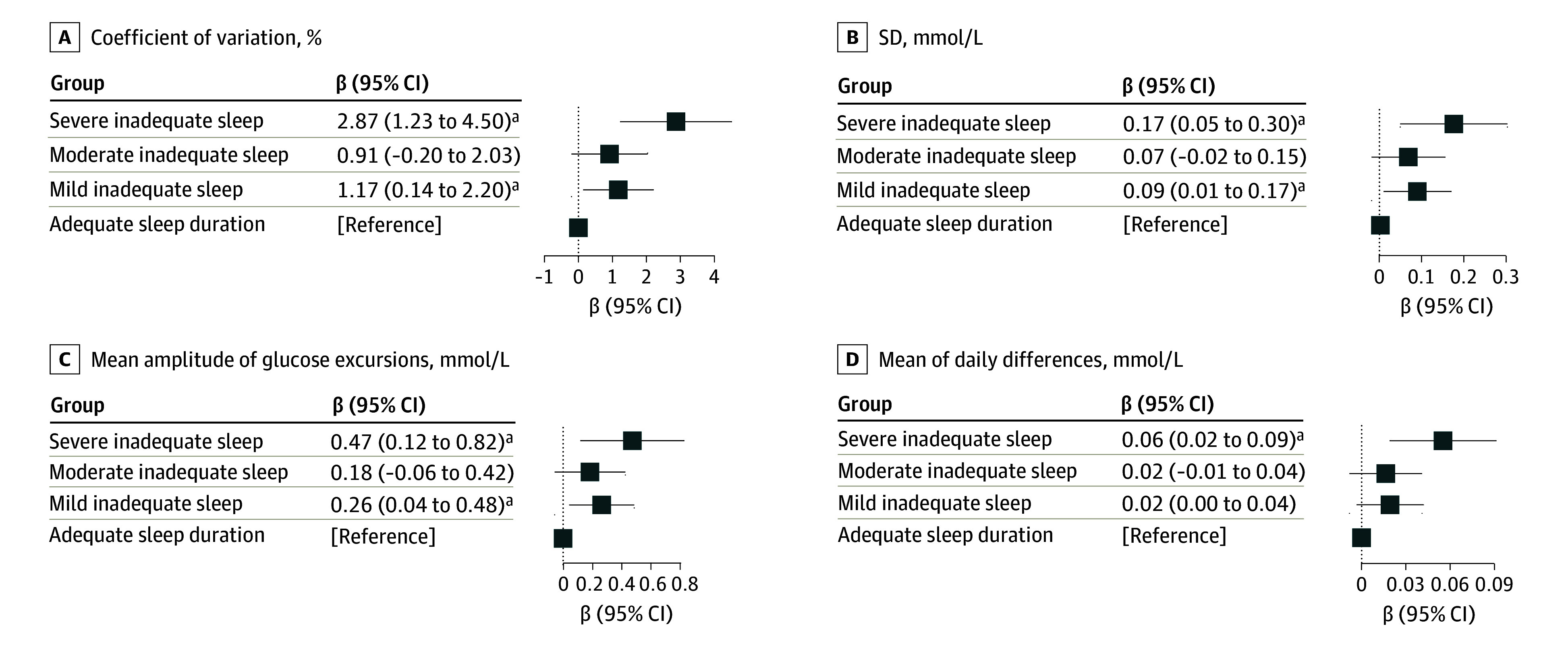
Association of the Sleep Duration Trajectories With Continuous Glucose Monitoring–Derived Metrics Association of long-term sleep duration patterns with glycemic variability, as qualified by coefficient of variation, SD, mean amplitude of glucose excursions, and mean of daily differences. β coefficients (95% CI) were derived from Huber robust regression models for sleep duration trajectories, severe inadequate sleep, moderate inadequate sleep, and mild inadequate sleep vs adequate sleep. Covariates included age, sex, body mass index, total energy intake, physical activity, income, education, smoking, alcohol drinking, and tea and coffee consumption. Multiple comparisons were controlled by false discovery rate of 0.05. Coefficient of variation was measured in percentage and SD, mean amplit ude, and mean of daily differences were measured in mmol/L. SI conversion factor: To convert glucose to mg/dL, divide by 0.0555. ^a^False discovery rate < 0.05.

We found that the trajectories characterized by inadequate sleep duration were positively associated with glycemic variability (CV, SD, MAGE, and MODD), and negatively associated with TIR (Figure 2; eFigure 3 and eTable 3 in Supplement 1). Compared with the adequate sleep duration trajectory, mild and severe inadequate sleep trajectory groups had increments of 1.17% (95% CI, 0.14%-2.20%), and 2.87% (95% CI, 1.23%-4.50%) CV, respectively (Figure 2A). Similarly, these groups also showed significant positive associations with SD (mild: β = 0.09 mmol/L; 95% CI, 0.01-0.17 mmol/L; severe: β = 0.17 mmol/L; 95% CI, 0.05-0.30 mmol/L) (Figure 2B) and MAGE (mild: β = 0.26 mmol/L; 95% CI, 0.04-0.48 mmol/L; severe: β = 0.47 mmol/L; 95% CI, 0.12-0.82 mmol/L) (Figure 2C), when compared with the adequate group. The severe inadequate sleep trajectory group also exhibited a higher MODD by 0.06 (95% CI, 0.02-0.09) mmol/L (Figure 2D).

In terms of mean glucose measures, the severe inadequate sleep duration group showed a lower TIR (β = −3.11%; 95% CI, −5.42% to −0.80%) compared with the adequate group (eFigure 3 in Supplement 1). These associations were consistent across models 1 through 3 (eTable 4 in Supplement 1). Sensitivity analyses confirmed the robustness of these findings, with no substantial alterations observed (eTable 5-eTable 7 in Supplement 1). No significant interactions were observed between sleep duration trajectories and age, sex, or BMI on any CGM-derived metrics.

### Associations Between Sleep Onset Timing Trajectories and CGM-Derived Glycemic Metrics

To evaluate the association between sleep onset timing and glycemic markers, we first identified 2 distinct trajectories of sleep onset timing: persistent early sleep onset group (878 participants [79.2%]), and persistently late sleep onset group (231 participants [20.8%]) (eFigure 4 in Supplement 1). Participants assigned to the persistently late sleep onset trajectory had significantly greater glycemic variability, as quantified by CV (β = 1.18%; 95% CI, 0.36%-2.01%), SD (β = 0.08 mmol/L; 95% CI, 0.01-0.14 mmol/L), and MODD (β = 0.02 mmol/L; 95% CI 0.01-0.04 mmol/L) (Table 2; eTable 8 and eFigure 5 in Supplement 1). These associations remained significant across models 1 through 3 (Table 2). The robustness of these findings was validated by conducting several sensitivity analyses, which yielded similar results as those presented in the main findings (eTables 9-10 in Supplement 1). Furthermore, there were no significant interaction observed between sleep onset trajectories and age, sex, or BMI on any CGM-derived metrics.

**Table 2.** Associations Between Sleep Onset Timing Trajectories and Continuous Glucose Monitoring-Derived Metrics^a^

Metric	Model 1^b^		Model 2^c^		Model 3^d^	
	β (95% CI)	*P* value	β (95% CI)	*P* value	β (95% CI)	*P* value
Glycemic variability						
CV, %	1.18 (0.35 to 2.01)	.01	1.18 (0.36 to 2.01)	.01	1.36 (0.44 to 2.28)	.004
SD, mmol/L	0.08 (0.02 to 0.15)	.01	0.08 (0.01 to 0.14)	.02	0.09 (0.02 to 0.15)	.01
MAGE, mmol/L	0.15 (−0.02 to 0.32)	.09	0.13 (−0.04 to 0.31)	.14	0.18 (0.00 to 0.36)	.06
MODD, mmol/L	0.03 (0.01 to 0.04)	.002	0.02 (0.01 to 0.04)	.01	0.02 (0.00 to 0.04)	.03
Mean glucose measures						
TIR, %	−1.02 (−2.19 to 0.15)	.09	−0.80 (−1.97 to 0.37)	.18	−0.63 (−1.95 to 0.69)	.35
MBG, mmol/L	0.07 (−0.04 to 0.18)	.21	0.07 (−0.05 to 0.18)	.24	0.09 (−0.03 to 0.21)	.15

Abbreviations: CV, coefficient of variation; MAGE, mean amplitude of glucose excursions; MBG, mean blood glucose; MODD, mean of daily differences; TIR, time in range.

SI conversion factor: To convert glucose to mg/dL, divide by 0.0555.

^a^ All values represent the associations between late sleep onset and CGM-derived metrics, with early sleep onset serving as the reference group.

^b^ Model 1: adjusted for age and sex.

^c^ Model 2: adjusted for age, sex, body mass index, physical activity, energy intake, education, income, smoke, alcohol, and tea and coffee.

^d^ Model 3: adjusted for age, sex, body mass index, physical activity, energy intake, education, income, smoke, alcohol, tea and coffee, and hemoglobin A_1c_ at the time when first sleep onset timing assessed.

### Joint Associations of Sleep Duration and Sleep Onset Timing Trajectories With Glycemic Variability

We next considered the combination of sleep duration and sleep onset trajectories for further analysis. A total of 26 participants (2.5%) were classified as having severe inadequate sleep duration with persistent late sleep onset, 32 (3.2%) as severe inadequate sleep duration with persistent early sleep onset, 181 (17.2%) as mild or moderate inadequate sleep duration with persistent late sleep onset, 698 (66.2%) as mild or moderate inadequate sleep duration with persistent early sleep onset, 12 (1.1%) as adequate sleep duration with persistent late sleep onset, and 104 (9.9%) as adequate sleep duration with persistent early sleep onset.

**Figure 3.**
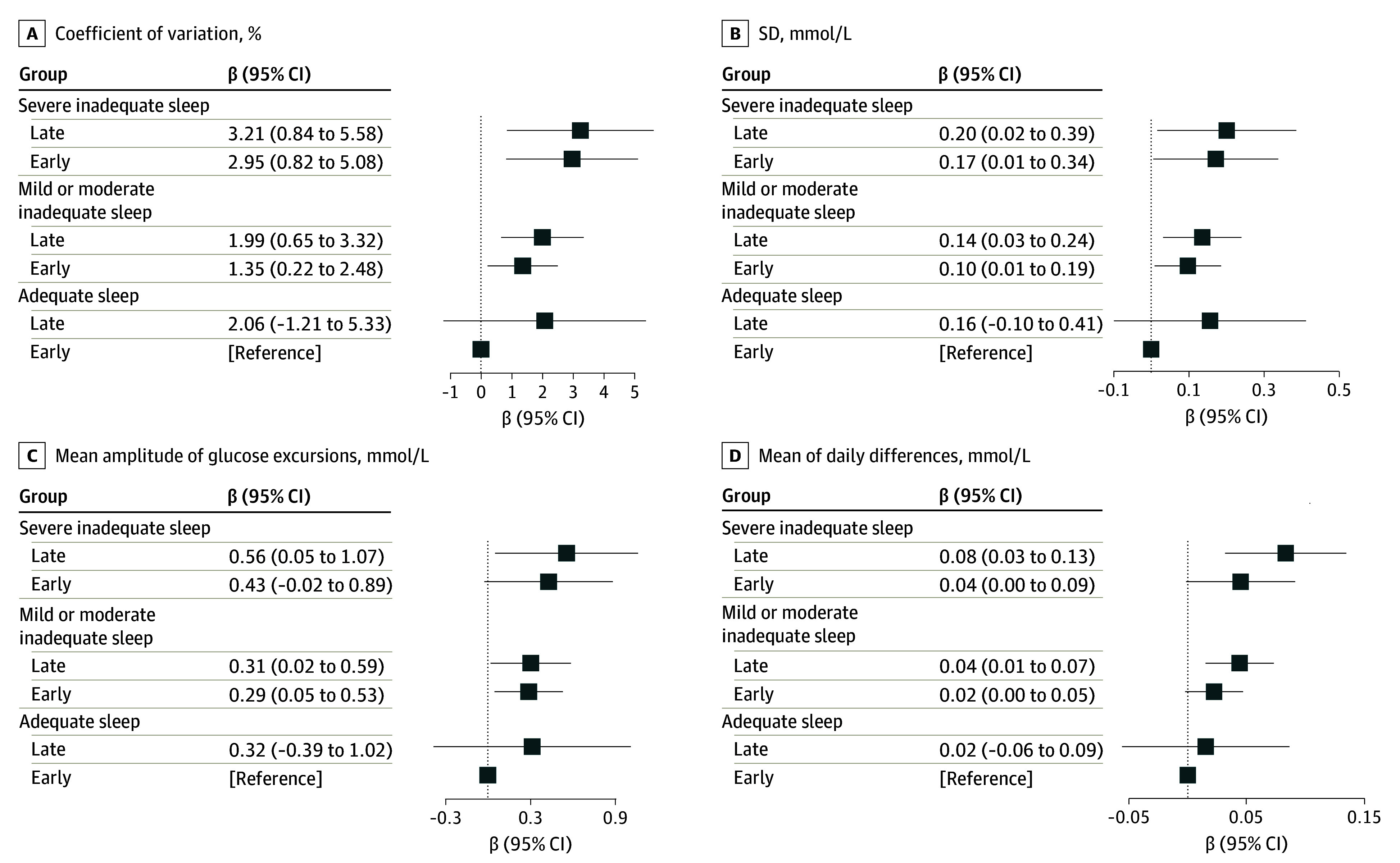
Joint Associations of Long-Term Sleep Duration Trajectories and Sleep Onset Timing Trajectories With Glycemic Variability Joint association of sleep patterns with glycemic variability, as qualified by CV, SD, MAGE, and MODD. Covariates included age, sex, body mass index, total energy intake, physical activity, income, education, smoking, alcohol drinking, and tea and coffee consumption. SI conversion factor: To convert glucose to mg/dL, divide by 0.0555. CV indicates coefficient of variation; MAGE, mean amplitude of glucose excursions; MODD, mean of daily differences.

Compared with individuals with adequate sleep and early sleep onset, combined exposure to inadequate sleep and late sleep onset was associated with elevated levels of glycemic variability, as evidenced by larger CV, SD, MAGE, and MODD (Figure 3). Within the strata of severe, mild or moderate inadequate, and adequate sleep duration groups, CV was always larger for the late sleep onset group compared with the early sleep onset group (Figure 3A). Between the strata of sleep duration, CV increased by 1.35% (95% CI, 0.22%-2.48%) for mild or moderate inadequate to early onset, and 2.95% (95% CI, 0.82%-5.08%) for severe inadequate to early onset, respectively, compared with the reference group of adequate to early onset (Figure 3A). The results for other glycemic variability measures led to similar conclusions (Figure 3B, 3C, and 3D).

## Discussion

This study profiled the association between long-term poor sleeping trajectories and glucose dynamics in the general population. Our findings revealed that suboptimal sleep trajectories, inadequate sleep duration, and improper timing of sleep, whether alone or in combination, exhibited larger glycemic variability.

Glycemic variability has been reported as an important risk factor for morbidity^5,22^ and mortality.^6,9^ Previous cross-sectional studies^11,12,22,23^ showed inconsistent findings on the association between sleep features with glycemic variability. One study^11^ based on a population without diabetes found sleep duration correlated only with MODD, while another study^22^ did not find any significant association. A study^12^ involving 28 patients with T2D identified an association between sleep duration and glucose CV. The inconsistency of these studies may rise from the misclassification of sleep patterns due to a single time point measurement and from potential reverse causality inherent in cross-sectional design. Our longitudinal study addressed the previously described limitations by leveraging unique data to track sleep duration trajectories over 6 years in over 1100 participants.

Interestingly, our study observed that both severe and mild inadequate sleep were associated with higher glycemic variability compared with adequate sleep, although the association was not statistically significant in the moderate inadequate sleep group. This discrepancy may be attributed to the smaller sample size of the moderate inadequate sleep group compared with the mild group, which reduced the statistical power to detect significant differences. Furthermore, uncontrolled confounding variables, such as stress, sleep irregularity, and vasomotor symptoms, may obscure true associations in the moderate group.

Beyond the association between sleep duration and glycemic control, our findings also revealed associations between postmidnight sleep onset and increased measures of glycemic variability. These observations were supported by a cross-sectional study^12^ involving 28 patients with T2D indicating that a delayed sleep midpoint was associated with elevated glucose SD during sleep. Our longitudinal study with a general population demonstrated that sleep after midnight was associated with not only increased SD, but also CV and MODD. Our findings emphasize the potential detrimental role of circadian rhythm misalignment on glycemic regulation, providing evidence to incorporate sleep timing into glucose management guidelines.

Our study goes beyond prior research by examining the joint associations of sleep duration and sleep onset timing with CGM-derived profiles, which is, to our knowledge, novel compared with the literature.^11,22^ While the joint association for glycemic dynamics has yet to be investigated, evidence links late sleep onset time and sleep duration with higher cardiometabolic risks.^24^ In the current study, we found that participants with this combined phenotype exhibited the greatest glycemic variability, suggesting that sleep onset time and duration could be promising factors in improving glucose metabolism. These factors could serve as low-cost and noninvasive interventions in the primary prevention of diabetes. Furthermore, our findings suggest that the benefits of early sleeping for glycemic control are robust and independent of sleep duration. This suggests that individuals who already achieve adequate sleep duration, or those who are subject to sleep time restriction, could improve glycemic regulation by adopting an earlier sleep time.

The association between the poor sleep status and impaired glucose homeostasis is biologically plausible.^2^ Short sleep duration could influence glucose responses by modulating sympathetic activation and cortisol levels.^25,26,27,28^ Chronic sleep insufficiency can activate inflammatory pathways, exacerbating glycemic abnormalities.^29,30,31,32^ Growth hormone, which is sleep dependent, also affects glucose metabolism.^33,34^ Delayed sleep onset disrupts the circadian rhythm, affecting glycemic health by causing shifts in hormone release patterns like cortisol and melatonin, and increasing proinflammatory cytokines.^35,36^ Further studies are warranted to unravel the intricate ways in which sleep duration and onset time affect glycemic dynamics.

### Strengths and Limitations

Our study has several strengths. First, we leveraged unique longitudinal sleep data to track sleep trajectories over 6 years, allowing us to understand how sleep patterns evolve in middle-aged and older Chinese adults. This method also mitigates misclassification from single-time point measurements and better captures the relationship between long-term poor sleep and glycemic dynamics. Second, CGM-derived metrics provide a more comprehensive evaluation of blood glucose homeostasis^14,15,16,17^ over consecutive 14-day periods. Lastly, our investigation considered the combined association of sleep duration and sleep onset timing with glycemic variability, which is rarely reported in previous studies.

Our study also has several limitations. First, sleep patterns were assessed via self-report rather than objective measures, though self-reported sleep status is widely used in previous research with clinical relevance.^21^ Second, factors such as sleep apnea were not thoroughly examined, which could potentially introduce bias. Third, the trajectory groups were based on limited measurements, potentially missing detailed chronic sleep fluctuations. More repeated measurements and longer follow-up are needed for comprehensive exploration. Fourth, while the combination of poor sleep and late sleep onset timing is associated with significantly larger glucose fluctuations, the small sample size of the adequate sleep duration with persistent late sleep onset group limits the reliability and generalizability of these findings, suggesting that they should be interpreted with caution. Further studies with larger sample sizes are warranted to validate these findings across different populations. Fifth, our sample is limited to middle-aged and elderly Chinese individuals, restricting the generalizability of our findings to other age groups or ethnicities. Additionally, residual confounding may not be entirely ruled out.

## Conclusions

In this cohort study of middle-aged and older participants, insufficient sleep duration and late sleep onset, whether alone or in combination, were associated with elevated glycemic variability in the general population. These findings underscore the importance of ensuring sufficient sleep duration and adhering to an early sleep onset to optimize glycemic control and mitigate related health complications.

## Notes

Supplement 1.
eMethods.

eReferences.
**eTable 1.** Trajectory Modeling Fit Criteria **eTable 2.** Comparison of Baseline Characteristics Between Participants Included and Those Excluded in the Study **eTable 3.** Actual Value of CGM-Derived Metrics Across Different Sleep Duration Trajectory Groups **eTable 4.** Association of Sleep Duration Trajectories With CGM-Derived Metrics in Model 1, Model 2, and Model 3 **eTable 5.** Sensitivity Analysis for the Association of Sleep Duration Trajectories With CGM-Derived Metrics After Additional Adjustment for Sleep Onset Timing, Diseases Status, and Sleep Apnea Indices **eTable 6.** Sensitivity Analysis for the Association of Sleep Duration Trajectory With CGM-Derived Metrics Using Mild Inadequate Sleep Duration Group as Reference **eTable 7. **Prospective Association Between Baseline Sleep Duration and Follow-up CGM-Derived Metrics in Model 1, Model 2, and Model 3 **eTable 8.** Actual Value of CGM-Derived Metrics Across Different Sleep Onset Timing Trajectory Groups **eTable 9.** Sensitivity Analysis for the Associations Between Sleep Onset Timing Trajectories and CGM-Derived Metrics After Additionally Adjusted for Sleep Duration, Diseases Status, Respectively **eTable 10. **Prospective Associations Between First Follow-up Sleep Onset Timing and Second Follow-up CGM-Derived Metrics in Model 1, Model 2, and Model 3 **eFigure 1.** Flowchart of Analysis Sample Selection **eFigure 2. **Predicted Sleep Duration Trajectories at Baseline, First Follow-up, and Second Follow-up **eFigure 3.** Association of the Sleep Duration Trajectories With CGM-Derived Glycemic Control **eFigure 4.** Description of Sleep Onset Timing at First Follow-up, and Second Follow-up by Groups of Trajectories of Sleep Onset **eFigure 5.** Association of Sleep Onset Timing Trajectories With CGM-Derived Metrics in Model 2

Supplement 2.
Data Sharing Statement
