# Supplementary material for: Trajectories of Sleep Duration, Sleep Onset Timing, and Continuous Glucose Monitoring in Adults
Source: JAMA Netw Open. 2025 Mar 5;8(3):e250114. doi: 10.1001/jamanetworkopen.2025.0114 (PMC11883496; doi:10.1001/jamanetworkopen.2025.0114)
Supplement: Supplement 1. — eMethods. eReferences. eTable 1. Trajectory Modeling Fit Criteria eTable 2. Comparison of Baseline Characteristics Between Participants Included and Those Excluded in the Study eTable 3. Actual Value of CGM-Derived Metrics Across Different Sleep Duration Trajectory Groups eTable 4. Association of Sleep Duration Trajectories With CGM-Derived Metrics in Model 1, Model 2, and Model 3 eTable 5. Sensitivity Analysis for the Association of Sleep Duration Trajectories With CGM-Derived Metrics After Additional Adjustment for Sleep Onset Timing, Diseases Status, and Sleep Apnea Indices eTable 6. Sensitivity Analysis for the Association of Sleep Duration Trajectory With CGM-Derived Metrics Using Mild Inadequate Sleep Duration Group as Reference eTable 7. Prospective Association Between Baseline Sleep Duration and Follow-up CGM-Derived Metrics in Model 1, Model 2, and Model 3 eTable 8. Actual Value of CGM-Derived Metrics Across Different Sleep Onset Timing Trajectory Groups eTable 9. Sensitivity Analysis for the Associations Between Sleep Onset Timing Trajectories and CGM-Derived Metrics After Additionally Adjusted for Sleep Duration, Diseases Status, Respectively eTable 10. Prospective Associations Between First Follow-up Sleep Onset Timing and Second Follow-up CGM-Derived Metrics in Model 1, Model 2, and Model 3 eFigure 1. Flowchart of Analysis Sample Selection eFigure 2. Predicted Sleep Duration Trajectories at Baseline, First Follow-up, and Second Follow-up eFigure 3. Association of the Sleep Duration Trajectories With CGM-Derived Glycemic Control eFigure 4. Description of Sleep Onset Timing at First Follow-up, and Second Follow-up by Groups of Trajectories of Sleep Onset eFigure 5. Association of Sleep Onset Timing Trajectories With CGM-Derived Metrics in Model 2 [file jamanetwopen-e250114-s001.pdf]

Supplemental Online Content

Shen L, Li B, Gou W, et al Trajectories of sleep duration, sleep onset timing and continuous glucose monitoring in adults. *JAMA Netw Open*. 2025;8(3):e250114. doi:10.1001/jamanetworkopen.2025.0114

eMethods.

eReferences

eTable 1. Trajectory Modeling Fit Criteria

eTable 2. Comparison of Baseline Characteristics Between Participants Included and Those Excluded in the Study

eTable 3. Actual Value of CGM-Derived Metrics Across Different Sleep Duration Trajectory Groups

eTable 4. Association of Sleep Duration Trajectories With CGM-Derived Metrics in Model 1, Model 2, and Model 3

eTable 5. Sensitivity Analysis for the Association of Sleep Duration Trajectories With CGM-Derived Metrics After Additional Adjustment for Sleep Onset Timing, Diseases Status, and Sleep Apnea Indices

eTable 6. Sensitivity Analysis for the Association of Sleep Duration Trajectory With CGM-Derived Metrics Using Mild Inadequate Sleep Duration Group as Reference

eTable 7. Prospective Association Between Baseline Sleep Duration and Follow-up CGM-Derived Metrics in Model 1, Model 2, and Model 3

eTable 8. Actual Value of CGM-Derived Metrics Across Different Sleep Onset Timing Trajectory Groups

eTable 9. Sensitivity Analysis for the Associations Between Sleep Onset Timing Trajectories and CGM-Derived Metrics After Additionally Adjusted for Sleep Duration, Diseases Status, Respectively

eTable 10. Prospective Associations Between First Follow-up Sleep Onset Timing and Second Follow-up CGM-Derived Metrics in Model 1, Model 2, and Model 3

eFigure 1. Flowchart of Analysis Sample Selection

eFigure 2. Predicted Sleep Duration Trajectories at Baseline, First Follow-up, and Second Follow-up

eFigure 3. Association of the Sleep Duration Trajectories With CGM-Derived Glycemic Control

© 2025 Shen L et al. *JAMA Network Open*.

eFigure 4. Description of Sleep Onset Timing at First Follow-up, and Second Follow-up by Groups of Trajectories of Sleep Onset

eFigure 5. Association of Sleep Onset Timing Trajectories With CGM-Derived Metrics in Model 2

This supplemental material has been provided by the authors to give readers additional information about their work.

© 2025 Shen L et al. *JAMA Network Open*.

eMethods

Assessment of covariates

Demographic and lifestyle factors

Demographic and lifestyle information were collected by self-report at each visit using standardized questionnaires. Smoking status was dichotomized into “currently smoking” and “not currently smoking”. Alcohol consumption was classified as either “current alcohol drinkers” or “not current alcohol drinkers”. Physical activity was quantified in metabolic equivalent for task (MET) hours per day, derived from responses to a 19-item physical activity questionnaire[1]. Tea or coffee consumption was categorized as “current drinkers” or “not current drinkers”. Total energy intake was estimated from a 79-item validated food frequency questionnaire[2]. Baseline data on education and household income were obtained through face-to-face interviews, with education stratified into “no more than high school”, “some collage”, and “bachelor’s degree and above”. Household income was categorized into four groups: ≤ 500 Chinese Yuan/month/person, 500-1,500 Chinese Yuan/month/person, 1,500-3,000 Chinese Yuan/month/person, and >3,000 Chinese Yuan/month/person.

Anthropometric and Clinical Measures

Hight and weight were measurements were conducted by trained nurses, facilitating the calculation of body mass index (BMI) in kilogram per square meters. Fasting glucose level was determined using a Roche Cobas 8000 c702 automated analyzer (Roche Diagnostics GmbH, Shanghai, China) employing colorimetric methods. Glycated hemoglobin (HbA1c) was measured with the Bio-Rad D-10 Hemoglobin Testing System.

© 2025 Shen L et al. *JAMA Network Open*.

Type 2 diabetes (T2D) was ascertained based on fasting blood glucose ≥ 7.0 mmol/L, glycated hemoglobin (HbAlc) ≥ 6.5%, or the usage of diabetic medication, in accordance with T2D diagnosis criteria established by the American Diabetes Association[3]. Hypertension was defined by currently use of antihypertensive medication, systolic blood pressure (SBP) ≥140 mmHg, or diastolic blood pressure (DBP) ≥90 mmHg[4]. Dyslipidemia was characterized by total cholesterol (TC) ≥ 6.2 mmol/L, low-density lipoprotein cholesterol (LDL) ≥ 4.1 mmol/L, high-density lipoprotein cholesterol (HDL) < 1.0 mmol/L, triglycerides (TG) ≥ 2.3 mmol/L or self-report medical history[5].

eReferences

1. Liu B, Woo J, Tang N, Ng K, Ip R, Yu A. Assessment of total energy expenditure in a Chinese population by a physical activity questionnaire: examination of validity. *Int J Food Sci Nutr.* 2001;52(3):269-282.
2. Zhang CX, Ho SC. Validity and reproducibility of a food frequency Questionnaire among Chinese women in Guangdong province. *Asia Pac J Clin Nutr.* 2009;18(2):240-250.
3. Association AD. Diagnosis and classification of diabetes mellitus. *Diabetes Care.* 2011;34 Suppl 1(Suppl 1):S62-69.
4. Chalmers J, MacMahon S, Mancia G, et al. 1999 World Health Organization-International Society of Hypertension Guidelines for the management of hypertension. Guidelines sub-committee of the World Health Organization. *Clin Exp Hypertens.* 1999;21(5-6):1009-1060.
5. Wibberley C. Seismology: talc at fault. *Nature.* 2007;448(7155):756-757.

© 2025 Shen L et al. *JAMA Network Open*.

eTable 1. Trajectory Modeling Fit Criteria

sleep duration					
	n (%)	posterior predictive probability			
		mean	SD	minimum	maximum
severe inadequate sleep	66 (5.7)	0.85	0.16	0.51	1.00
moderate inadequate sleep	316 (27.3)	0.76	0.12	0.52	0.94
mild inadequate sleep	641 (55.4)	0.77	0.11	0.50	0.91
adequate	113 (11.5)	0.74	0.16	0.50	1.00
sleep onset timing					
	n (%)	posterior predictive probability			
		mean	SD	minimum	maximum
persistent early sleep onset	878 (79.2)	0.94	0.11	0.51	1.00
persistent late sleep onset	231 (20.8)	0.85	0.16	0.51	1.00

© 2025 Shen L et al. *JAMA Network Open*.

eTable 2. Comparison of Baseline Characteristics Between Participants Included and Those Excluded in the Study

Characteristics	**Include (n=1156)**	**Exclude (n=1378)**
Age, years, mean (SD)	63.0 (5.1)	63.6 (5.3)
Men, n (%)	340 (29.4)	414 (30)
BMI, kg/m2, mean (SD)	23.4 (3.3)	23.8 (3.3)
Current smoker, n (%)	63 (5.4)	134 (9.7)
Current alcohol drinker, n (%)	97 (8.4)	108 (7.8)
Tea consumption, n (%)	600 (51.9)	695 (50.6)
Education, n (%)		
Middle school or lower	266 (23)	410 (29.8)
High school or professional college	541 (46.8)	656 (47.7)
University	349 (30.2)	308 (22.4)
Household income (Chinese Yuan/month/person), n (%)		
Extremely Low (≤500 ¥/month)	21 (1.8)	34 (2.5)
Low (501-1500 ¥/month)	276 (23.9)	436 (31.7)
Middle (1501-3000 ¥/month)	539 (46.6)	649 (47.2)
High (>3000 ¥/month)	320 (27.7)	255 (18.6)
Physical activity, MET-h/day, mean (SD)	42.1 (15.1)	42.5 (15.5)
Total energy intake, kcal/day, mean (SD)	1800.8 (564.4)	1858.5 (635)

Abbreviations: BMI, body mass index; MET, metabolic equivalent of task.

© 2025 Shen L et al. *JAMA Network Open*.

eTable 3. Actual Value of CGM-Derived Metrics Across Different Sleep Duration Trajectory Groups

CGM-derived metrics	sleep duration			
	severe inadequate sleep	moderate inadequate sleep	mild inadequate sleep	adequate sleep
CV, %, mean (SD)	25.42 (5.87)	24.19 (5.80)	24.47 (5.64)	23.42 (5.88)
SD, mmol/L, mean (SD)	1.54 (0.59)	1.45 (0.54)	1.47 (0.55)	1.38 (0.49)
MAGE, mmol/L, mean (SD)	3.76 (1.41)	3.52 (1.27)	3.62 (1.35)	3.37 (1.26)
MODD, mmol/L, median (IQR)	0.28 (0.21)	0.24 (0.15)	0.25 (0.16)	0.22 (0.14)
TIR, %, median (IQR)	92.62 (14.07)	94.14 (10.85)	93.66 (11.02)	95.49 (10.76)
daily MBG, mmol/L, mean (SD)	5.98 (1.35)	5.93 (1.38)	5.96 (1.45)	5.83 (0.97)

Abbreviations: CGM, continuous glucose monitoring; MBG, mean blood glucose; SD, standard deviation; CV, coefficient of variation; MAGE, mean amplitude of glycemic excursions; MODD, mean of daily differences; TIR, time in range.

© 2025 Shen L et al. *JAMA Network Open*.

eTable 4. Association of Sleep Duration Trajectories With CGM-Derived Metrics in Model 1, Model 2, and Model 3

		model 1		model 2		model 3	
		beta (95%CI)	*P*	beta (95%CI)	*P*	beta (95%CI)	*P*
Glycemic variability							
SD	severe inadequate	0.14 (0.01, 0.27)	**0.03**	0.17 (0.05, 0.30)	**0.007**	0.14 (0.02,0.26)	**0.03**
	moderate inadequate	0.05 (-0.04, 0.14)	0.24	0.07 (-0.02, 0.15)	0.14	0.06 (-0.02,0.14)	0.16
	mild inadequate	0.07 (-0.01, 0.16)	0.07	0.09 (0.01, 0.17)	**0.03**	0.08 (0.00,0.15)	**0.05**
	adequate	ref		ref		ref	
CV	severe inadequate	2.51 (0.88, 4.15)	**0.003**	2.87 (1.23, 4.50)	**0.0006**	2.45 (0.85,4.05)	**0.003**
	moderate inadequate	0.92 (-0.21, 2.04)	0.11	0.91 (-0.20, 2.03)	0.11	0.81 (-0.29,1.90)	0.15
	mild inadequate	1.19 (0.15, 2.22)	**0.02**	1.17 (0.14, 2.20)	**0.03**	1.00 (-0.01,2.00)	**0.05**
	adequate	ref		ref		ref	
MAGE	severe inadequate	0.40 (0.04, 0.75)	**0.03**	0.47 (0.12, 0.82)	**0.008**	0.39 (0.05,0.72)	**0.02**
	moderate inadequate	0.16 (-0.09, 0.40)	0.21	0.18 (-0.06, 0.42)	0.14	0.17 (-0.06,0.40)	0.14
	mild inadequate	0.23 (0.01, 0.46)	**0.04**	0.26 (0.04, 0.48)	**0.02**	0.25 (0.04,0.46)	**0.02**
	adequate	ref		ref		ref	
MODD	severe inadequate	0.05 (0.01, 0.08)	**0.007**	0.06 (0.02, 0.09)	**0.003**	0.05 (0.01,0.08)	**0.006**
	moderate inadequate	0.01 (-0.01, 0.03)	0.4	0.02 (-0.01, 0.04)	0.19	0.02 (0.00,0.05)	0.08
	mild inadequate	0.02 (-0.01, 0.04)	0.19	0.02 (0.00, 0.04)	0.1	0.02 (0.00,0.04)	0.06
	adequate	ref		ref		ref	
Mean glucose measures							
TIR	severe inadequate	-2.77 (-5.07, -0.46)	**0.02**	-3.11 (-5.42, -0.8)	**0.008**	-3.04 (-5.31,-0.77)	**0.009**
	moderate inadequate	-0.75 (-2.33, 0.83)	0.35	-0.83 (-2.41, 0.75)	0.30	-1.07 (-2.63,0.48)	0.18
	mild inadequate	-0.93 (-2.39, 0.52)	0.21	-0.88 (-2.34, 0.58)	0.24	-1.14 (-2.57,0.29)	0.12
	adequate	ref		ref		ref	
MBG	severe inadequate	0.04 (-0.19, 0.26)	0.75	0.10 (-0.13, 0.32)	0.41	0.05 (-0.17,0.27)	0.65

© 2025 Shen L et al. *JAMA Network Open*.

moderate inadequate	-0.02 (-0.18, 0.13)	0.76	0.01 (-0.15, 0.16)	0.93	0.01 (-0.14,0.15)	0.93
mild inadequate	0.02 (-0.12, 0.16)	0.79	0.05 (-0.09, 0.19)	0.49	0.04 (-0.09,0.18)	0.54
adequate	ref		ref		ref	

Abbreviations: CGM, continuous glucose monitoring; MBG, mean blood glucose; SD, standard deviation; CV, coefficient of variation; MAGE, mean amplitude of glycemic excursions; MODD, mean of daily differences; TIR, time in range.

Model 1: adjusted for age, sex.

Model 2: adjusted for age, sex, BMI, physical activity, energy intake, education, income, smoke, alcohol, tea and coffee.

Model 3: adjusted for age, sex, BMI, physical activity, energy intake, education, income, smoke, alcohol, tea and coffee, and HbA1c at baseline.

© 2025 Shen L et al. *JAMA Network Open*.

eTable 5. Sensitivity Analysis for the Association of Sleep Duration Trajectories With CGM-Derived Metrics After Additional Adjustment for Sleep Onset Timing, Diseases Status, and Sleep Apnea Indices

		model 1		model 2		model 3	
		beta (95%CI)	*P*	beta (95%CI)	*P*	beta (95%CI)	*P*
Glycemic variability							
SD	severe inadequate	0.14 (0.01,0.28)	**0.04**	0.14 (0.01,0.26)	**0.03**	0.13(0, 0.26)	**0.04**
	moderate inadequate	0.05 (-0.04,0.14)	0.30	0.05 (-0.04,0.13)	0.27	0.03(-0.06, 0.12)	0.47
	mild inadequate	0.09 (0.01,0.17)	**0.04**	0.07 (-0.0,0.15)	0.06	0.07(-0.01, 0.16)	0.08
	adequate	ref		ref		ref	
CV	severe inadequate	2.43 (0.68,4.18)	**0.006**	2.62 (1.05,4.19)	**0.001**	2.59 (0.92, 4.26)	**0.002**
	moderate inadequate	0.70 (-0.49,1.89)	0.25	0.67 (-0.40,1.75)	0.22	0.50 (-0.66, 1.65)	0.40
	mild inadequate	1.24 (0.15,2.34)	**0.03**	0.95 (-0.04,1.94)	0.06	1.05 (-0.01, 2.11)	**0.05**
	adequate	ref		ref		ref	
MAGE	severe inadequate	0.40 (0.02,0.77)	**0.04**	0.40 (0.06,0.73)	**0.02**	0.39 (0.04, 0.75)	**0.03**
	moderate inadequate	0.15 (-0.11,0.40)	0.26	0.14 (-0.09,0.37)	0.22	0.11 (-0.14, 0.35)	0.39
	mild inadequate	0.27 (0.04,0.51)	**0.02**	0.24 (0.03,0.45)	**0.02**	0.25 (0.03, 0.48)	**0.03**
	adequate	ref		ref		ref	
MODD	severe inadequate	0.05 (0.01,0.09)	**0.01**	0.05 (0.01,0.08)	**0.006**	0.05 (0.01, 0.08)	**0.008**
	moderate inadequate	0.01 (-0.01,0.04)	0.30	0.02 (-0.00,0.04)	0.09	0.02 (-0.01, 0.04)	0.18
	mild inadequate	0.02 (-0.00,0.05)	0.06	0.02 (-0.00,0.04)	0.08	0.02 (0, 0.04)	0.09
	adequate	ref		ref		ref	
Mean glucose measures							
TIR	severe inadequate	-2.73 (-5.22,-0.25)	**0.03**	-2.68 (-4.9,-0.46)	**0.02**	-2.8 (-5.15, -0.44)	**0.02**
	moderate inadequate	-0.82 (-2.51,0.87)	0.34	-0.8 (-2.32,0.72)	0.30	-0.75 (-2.38, 0.88)	0.37
	mild inadequate	-1.14 (-2.69,0.42)	0.15	-0.96 (-2.36,0.44)	0.18	-1.15 (-2.65, 0.34)	0.13
	adequate	ref		ref		ref	

© 2025 Shen L et al. *JAMA Network Open*.

Abbreviations: CGM, continuous glucose monitoring; MBG, mean blood glucose; SD, standard deviation; CV, coefficient of variation; MAGE, mean amplitude of glycemic excursions; MODD, mean of daily differences; TIR, time in range.

Model 1: adjusted for age, sex, BMI, physical activity, energy intake, education, income, smoke, alcohol, tea, and sleep onset timing.

Model 2: adjusted for age, sex, BMI, physical activity, energy intake, education, income, smoke, alcohol, tea, type 2 diabetes, hypertension, and dyslipidemia.

Model 3: adjusted for age, sex, BMI, physical activity, energy intake, education, income, smoke, alcohol, tea, snoring, sleepy, type 2 diabetes, hypertension, and dyslipidemia.

© 2025 Shen L et al. *JAMA Network Open*.

eTable 6. Sensitivity Analysis for the Association of Sleep Duration Trajectory With CGM-Derived Metrics Using Mild Inadequate Sleep Duration Group as Reference

		model	
		beta(95%CI)	*P*
Glycemic variability			
SD	severe inadequate	0.09(-0.02,0.20)	0.12
	moderate inadequate	-0.02(-0.08,0.04)	0.47
	adequate	-0.09(-0.17,-0.01)	**0.03**
	mild inadequate	ref	
CV	severe inadequate	1.70(0.29,3.10)	**0.02**
	moderate inadequate	-0.26(-1.00,0.49)	0.50
	adequate	-1.17(-2.20,-0.14)	**0.03**
	mild inadequate	ref	
MAGE	severe inadequate	0.21(-0.09,0.51)	0.18
	moderate inadequate	-0.08(-0.24,0.08)	0.33
	adequate	-0.26(-0.48,-0.04)	**0.02**
	mild inadequate	ref	
MODD	severe inadequate	0.04(0.01,0.07)	**0.02**
	moderate inadequate	0.00(-0.02,0.01)	0.73
	adequate	-0.02(-0.04,0.00)	0.10
	mild inadequate	ref	
Mean glucose measures			
TIR	severe inadequate	-2.23(-4.22,-0.24)	**0.03**
	moderate inadequate	0.05(-1.00,1.10)	0.92
	adequate	0.88(-0.58,2.34)	0.24
	mild inadequate	ref	

Abbreviations: CGM, continuous glucose monitoring; MBG, mean blood glucose; SD, standard deviation; CV, coefficient of variation; MAGE, mean amplitude of glycemic excursions; MODD, mean of daily differences; TIR, time in range.

© 2025 Shen L et al. *JAMA Network Open*.

eTable 7. Prospective Association Between Baseline Sleep Duration and Follow-up CGM-Derived Metrics in Model 1, Model 2, and Model 3

		model 1			model 2			model 3		
		beta (95%CI)	*P*	*P-trend*	beta (95%CI)	*P*	*P-trend*	beta (95%CI)	*P*	*P-trend*
Glycemic variability										
SD	severe inadequate	0.10(0.00,0.19)	**0.05**	0.08	0.10(0.01,0.20)	**0.03**	**0.04**	0.09(-0.01,0.18)	0.06	0.07
	moderate inadequate	0.09(-0.01,0.18)	0.07		0.10(0.01,0.20)	**0.04**		0.10(0.01,0.19)	**0.04**	
	mild inadequate	0.07(-0.03,0.17)	0.15		0.07(-0.03,0.17)	0.16		0.06(-0.03,0.16)	0.19	
	adequate	ref			ref			ref		
CV	severe inadequate	1.35(0.10,2.61)	**0.03**	**0.05**	1.33(0.07,2.60)	**0.04**	**0.05**	1.18(-0.06,2.42)	0.06	0.09
	moderate inadequate	0.81(-0.44,2.06)	0.21		0.89(-0.38,2.15)	0.17		0.81(-0.43,2.04)	0.20	
	mild inadequate	0.88(-0.40,2.15)	0.18		0.82(-0.46,2.11)	0.21		0.80(-0.45,2.05)	0.21	
	adequate	ref			ref			ref		
MAGE	severe inadequate	0.26(-0.01,0.52)	0.06	0.10	0.27(0.00,0.53)	**0.05**	0.08	0.25(-0.01,0.50)	0.06	0.10
	moderate inadequate	0.24(-0.03,0.50)	0.08		0.27(0.00,0.53)	**0.05**		0.27(0.02,0.52)	**0.03**	
	mild inadequate	0.19(-0.07,0.46)	0.15		0.20(-0.07,0.47)	0.16		0.20(-0.05,0.46)	0.12	
	adequate	ref			ref			ref		
MODD	severe inadequate	0.01(-0.02,0.03)	0.63	0.43	0.01(-0.02,0.04)	0.48	0.28	0.00(-0.02,0.03)	0.71	0.33
	moderate inadequate	0.01(-0.02,0.04)	0.5		0.01(-0.02,0.04)	0.45		0.00(-0.02,0.03)	0.74	
	mild inadequate	0.00(-0.03,0.03)	0.97		0.00(-0.03,0.03)	0.99		-0.01(-0.03,0.02)	0.62	
	adequate	ref			ref			ref		
Mean glucose measures										
TIR	severe inadequate	-0.54(-2.26,1.18)	0.54	0.40	-0.60(-2.38,1.18)	0.51	0.37	-0.64(-2.41,1.13)	0.48	0.29
	moderate inadequate	-0.31(-2.03,1.42)	0.73		-0.26(-2.05,1.52)	0.77		-0.49(-2.26,1.28)	0.59	
	mild inadequate	-0.06(-1.81,1.69)	0.95		-0.04(-1.84,1.77)	0.97		0.02(-1.77,1.82)	0.98	
	adequate	ref			ref			ref		

© 2025 Shen L et al. *JAMA Network Open*.

Abbreviations: CGM, continuous glucose monitoring; MBG, mean blood glucose; SD, standard deviation; CV, coefficient of variation; MAGE, mean amplitude of glycemic excursions; MODD, mean of daily differences; TIR, time in range.

Model 1: adjusted for age, sex.

Model 2: adjusted for age, sex, BMI, physical activity, energy intake, education, income, smoke, alcohol, tea.

Model 3: adjusted for age, sex, BMI, physical activity, energy intake, education, income, smoke, alcohol, tea, and HbA1c at baseline.

© 2025 Shen L et al. *JAMA Network Open*.

eTable 8. Actual Value of CGM-Derived Metrics Across Different Sleep Onset Timing Trajectory Groups

CGM-derived metrics	sleep onset timing	
	late onset	early onset
CV, %, mean (SD)	25.13 (6.13)	24.16 (5.66)
SD, mmol/L, mean (SD)	1.51 (0.57)	1.44 (0.52)
MAGE, mmol/L, mean (SD)	3.66 (1.39)	3.54 (1.30)
MODD, mmol/L, median (IQR)	0.25 (0.18)	0.24 (0.15)
TIR, %, median (IQR)	93.49 (13.19)	94.05 (11.11)
daily MBG, mmol/L, mean (SD)	5.94 (1.35)	5.90 (1.34)

Abbreviations: CGM, continuous glucose monitoring; MBG, mean blood glucose; SD, standard deviation; CV, coefficient of variation; MAGE, mean amplitude of glycemic excursions; MODD, mean of daily differences; TIR, time in range.

© 2025 Shen L et al. *JAMA Network Open*.

eTable 9. Sensitivity Analysis for the Associations Between Sleep Onset Timing Trajectories and CGM-Derived Metrics After Additionally Adjusted for Sleep Duration, Diseases Status, Respectively

		model 1		model 2	
		beta (95% CI)	*P*	beta (95% CI)	*P*
Glycemic variability					
CV	after midnight	0.07 (0,0.13)	**0.05**	0.07 (0.01,0.13)	**0.02**
	before midnight	ref		ref	
SD	after midnight	0.96 (0.12,1.8)	**0.02**	1.12 (0.32,1.92)	**0.006**
	before midnight	ref		ref	
MAGE	after midnight	0.08 (-0.1,0.27)	0.37	0.13 (-0.03,0.29)	0.12
	before midnight	ref		ref	
MODD	after midnight	0.02 (0.01,0.04)	**0.01**	0.02 (0,0.04)	**0.01**
	before midnight	ref		ref	

Abbreviations: CGM, continuous glucose monitoring; MBG, mean blood glucose; SD, standard deviation; CV, coefficient of variation; MAGE, mean amplitude of glycemic excursions; MODD, mean of daily differences; TIR, time in range.

Model 1: adjusted for age, sex, BMI, physical activity, energy intake, education, income, smoke, alcohol, tea, and sleep duration.

Model 2: adjusted for age, sex, BMI, physical activity, energy intake, education, income, smoke, alcohol, tea, type 2 diabetes, hypertension, and dyslipidemia.

© 2025 Shen L et al. *JAMA Network Open*.

eTable 10. Prospective Associations Between First Follow-up Sleep Onset Timing and Second Follow-up CGM-Derived Metrics in Model 1, Model 2, and Model 3

		model 1		model 2		model 3	
Glycemic variability		beta (95% CI)	*P*	beta (95% CI)	*P*	beta (95% CI)	*P*
SD	after midnight	0.06(0.00,0.12)	0.06	0.04(-0.02,0.11)	0.16	0.07(0.00,0.13)	**0.04**
	before midnight	ref		ref		ref	
CV	after midnight	0.76(-0.05,1.57)	0.07	0.70(-0.11,1.51)	0.09	0.96(0.05,1.86)	**0.04**
	before midnight	ref		ref		ref	
MAGE	after midnight	0.11(-0.06,0.28)	0.21	0.08(-0.09,0.25)	0.37	0.17(-0.01,0.35)	0.06
	before midnight	ref		ref		ref	
MODD	after midnight	0.02(0.01,0.04)	**0.005**	0.02(0.00,0.04)	**0.03**	0.02(0.00,0.04)	**0.05**
	before midnight	ref		ref		ref	

Abbreviations: CGM, continuous glucose monitoring; MBG, mean blood glucose; SD, standard deviation; CV, coefficient of variation; MAGE, mean amplitude of glycemic excursions; MODD, mean of daily differences; TIR, time in range.

Model 1: adjusted for age, sex.

Model 2: adjusted for age, sex, BMI, physical activity, energy intake, education, income, smoke, alcohol, tea.

Model 3: adjusted for age, sex, BMI, physical activity, energy intake, education, income, smoke, alcohol, tea, and HbA1c at baseline.

© 2025 Shen L et al. *JAMA Network Open*.

eFigure 1. Flowchart of Analysis Sample Selection

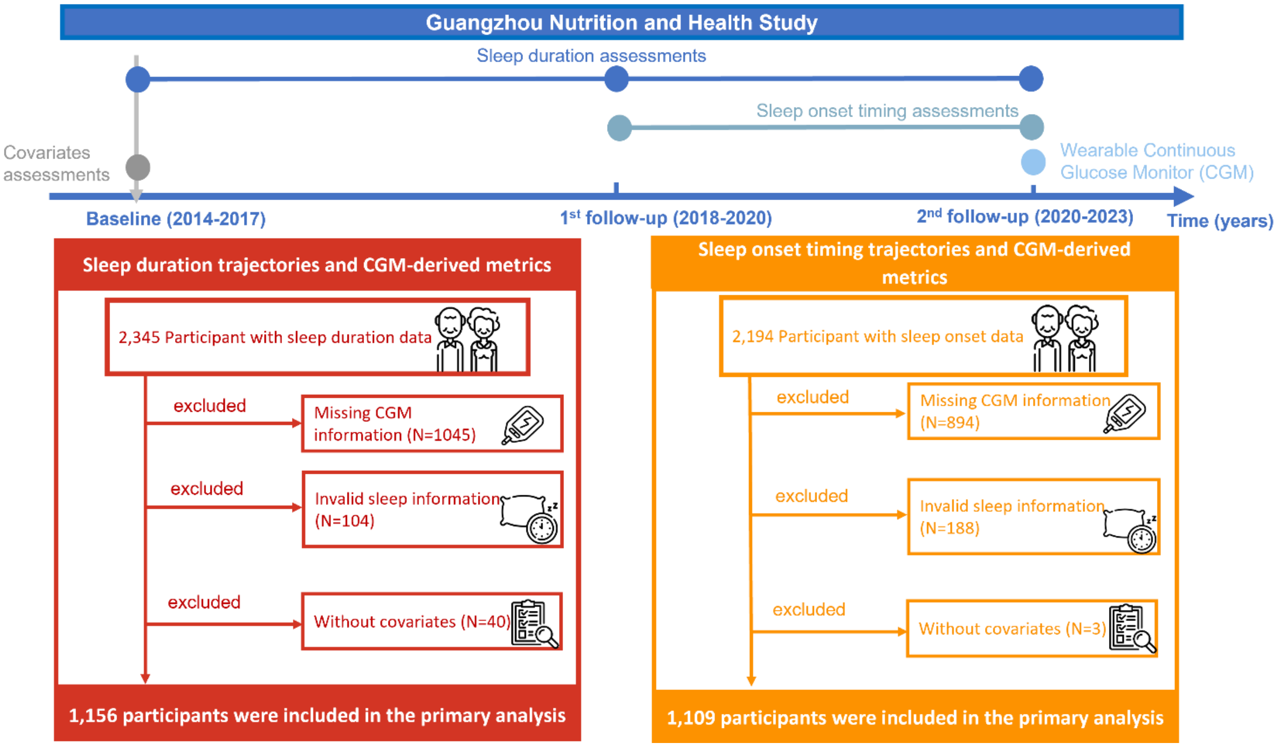

Abbreviations: GNHS, Guangzhou Nutrition and Health Study; CGM, continuous glucose monitoring.

© 2025 Shen L et al. *JAMA Network Open*.

eFigure 2. Predicted Sleep Duration Trajectories at Baseline, First Follow-up, and Second Follow-up

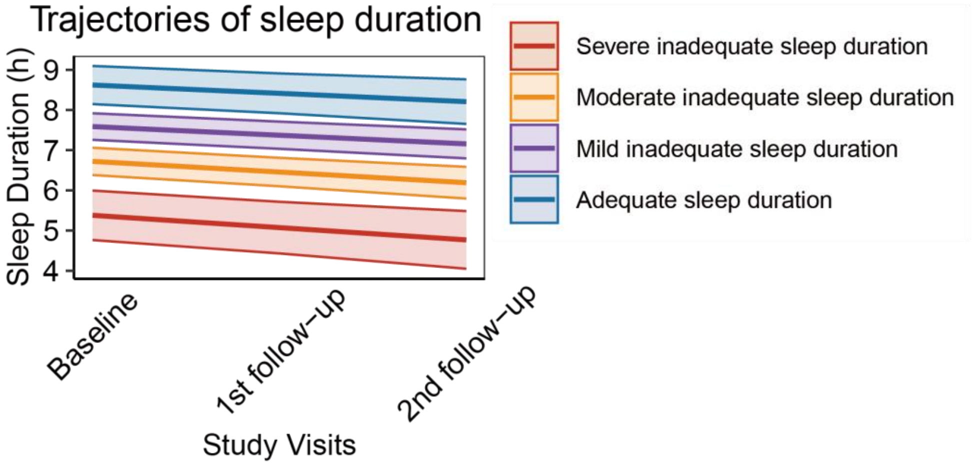

Sleep duration pattern	Sample size	Sleep duration, mean (standard deviation) (h)		
		Baseline	1st follow-up	2nd follow-up
Severe inadequate sleep duration	66	4.72 (0.82)	4.08 (0.74)	4.15 (0.85)
Moderate inadequate sleep duration	316	6.02 (0.90)	5.70 (0.81)	5.48 (0.83)
Mild inadequate sleep duration	641	7.18 (0.84)	6.91 (0.87)	6.76 (0.80)
Adequate sleep duration	133	8.42 (0.79)	8.19 (0.74)	8.02 (0.76)

© 2025 Shen L et al. *JAMA Network Open*.

eFigure 3. Association of the Sleep Duration Trajectories With CGM-Derived Glycemic Control

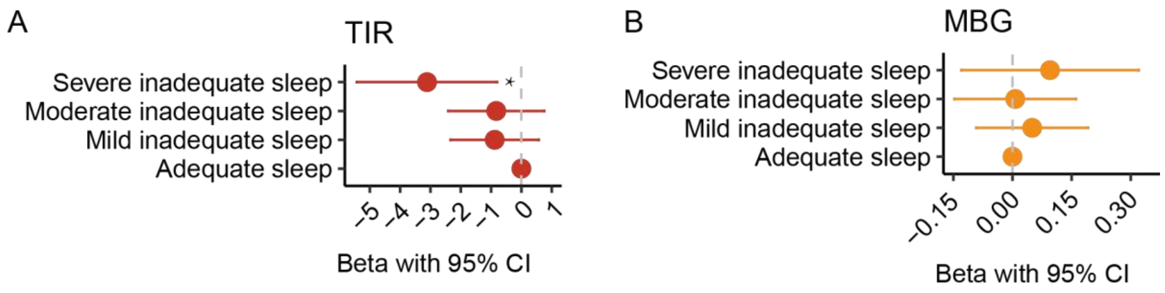

Association of long-term sleep duration patterns with glycemic variability, as qualified by TIR (**A**), MBG (**B**). Beta coefficients (95% CI) were derived from Huber robust regression models for sleep duration trajectories, severe inadequate sleep, moderate inadequate sleep, mild inadequate sleep vs. adequate sleep. Covariates included age, sex, BMI, total energy intake, physical activity, income, education, smoking, alcohol drinking, tea and coffee consumption. Multiple comparisons were controlled by false discovery rate (FDR < 0.05). The asterisks represent FDR<0.05. Abbreviations: CI, confidence interval; TIR, time in range; MBG, mean blood glucose.

© 2025 Shen L et al. *JAMA Network Open*.

eFigure 4. Description of Sleep Onset Timing at First Follow-up, and Second Follow-up by Groups of Trajectories of Sleep Onset

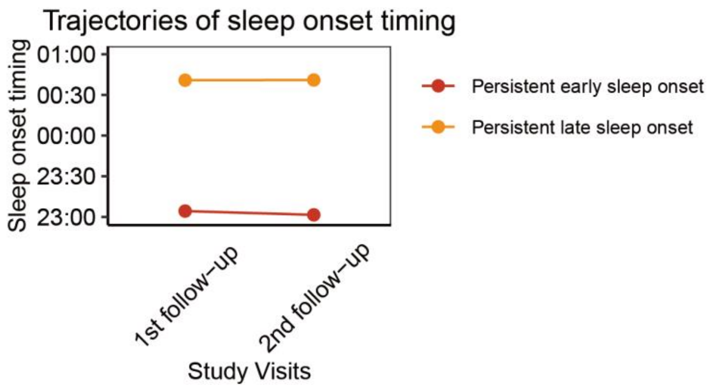

Sleep onset timing pattern	Sample size	Sleep duration, mean (standard deviation) (h)	
		1st follow-up	2nd follow-up
Persistent early sleep onset	878	23.07 (0.69)	23.02(0.72)
Persistent late sleep onset	231	24.68 (0.74)	24.68 (0.85)

© 2025 Shen L et al. *JAMA Network Open*.

eFigure 5. Association of Sleep Onset Timing Trajectories With CGM-Derived Metrics in Model 2

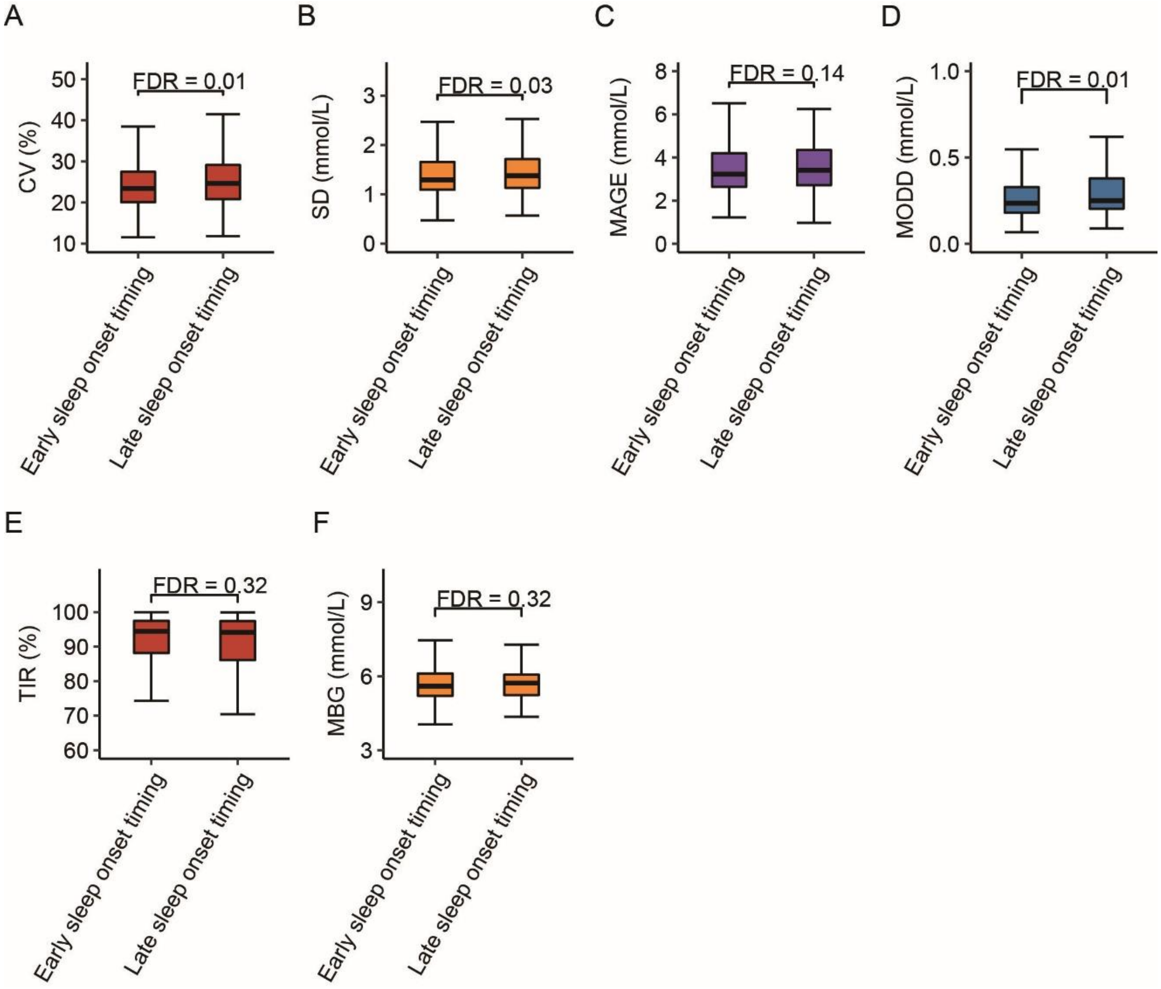

Box plots presents the median (interquartile range) of CV (A), SD (B), MAGE (C), MODD (D), TIR (E), MBG (F) for early sleep onset and late sleep onset group, respectively. Huber robust regression models were performed to examine the associations of sleep onset trajectories with CGM-derived measures (glycemic variability measures and mean-glucose measures), adjusting for covariates in model 2. False discovery rate (FDR) was applied to correct for multiple comparisons using the Benjamini-Hochberg method. Abbreviations: MBG, mean blood glucose; CV, coefficient of variation; SD,

© 2025 Shen L et al. *JAMA Network Open*.

standard deviation; MAGE, mean amplitude of glycemic excursions; MODD, mean of daily differences; TIR, time in range.

© 2025 Shen L et al. *JAMA Network Open*.
