# Supplementary material for: Trajectories of Sleep Duration, Sleep Onset Timing, and Continuous Glucose Monitoring in Adults
Source: JAMA Netw Open. 2025 Mar 5;8(3):e250114. doi: 10.1001/jamanetworkopen.2025.0114 (PMC11883496; doi:10.1001/jamanetworkopen.2025.0114)
Supplement: Supplement 2. — Data Sharing Statement [file jamanetwopen-e250114-s002.pdf]

Data Sharing Statement

Shen. Trajectories of Sleep Duration, Sleep Onset Timing and Continuous Glucose Monitoring in Adults. *JAMA Netw Open*. Published March 05, 2025.
doi:10.1001/jamanetworkopen.2025.0114

Data
Data available: No
